# WZY-321 triggers glioma cell apoptosis via XAF1 up-regulation caused by MTM-mediated miR-873 down-regulation

**DOI:** 10.7150/jca.68775

**Published:** 2022-04-18

**Authors:** Guan Sun, Wei Yuan, Weiye Zhu, Jian Chen

**Affiliations:** 1 Department of Neurosurgery, The Fourth Affiliated Hospital of Nantong University, The First People's Hospital of Yancheng, Yancheng, P.R. China; 2 Department of Neurosurgery, The Affiliated Hospital of Nantong University, Nantong, P.R. China

**Keywords:** gliomas, apoptosis, WZY-321, XAF1, miR-873, MTM

## Abstract

Gliomas account for the majority of primary malignant brain tumors around the world and are highly aggressive. Evodiamine is one of the main effective components of Evodia rutaecarpa, which can inhibit proliferation and promote apoptosis of tumor cells including glioma cells. The derivative of Evodiamine named WZY-321 was successfully developed, and exhibited significant cytotoxicity and could efficiently induce glioma cell apoptosis; however, the mechanism of WZY-321-induced glioma cell apoptosis is not clear. Our current studies showed that WZY-321 increased X-linked inhibitor of apoptosis-associated factor 1 (XAF1) expression in glioma cells, and up-regulated XAF1 resulted in glioma cell apoptosis. Moreover, WZY-321 treatment decreased miR-873 expression and increased lncRNA MTM expression in glioma cells, and down-regulated miR-873 or up-regulated MTM lead to glioma cell apoptosis. Mechanically, WZY-321 up-regulated XAF1 gene expression via MTM-decreased miR-873 expression, that bound to XAF1 3' UTR and decreased XAF1 mRNA levels. Taken together, these data indicate that WZY-321 triggers glioma cell apoptosis via XAF1 up-regulation caused by MTM-mediated miR-873 down-regulation.

## Introduction

Gliomas account for the majority of primary malignant brain tumors around the world and are highly aggressive 1-4. Gliomas usually originate from glial, choroid plexus epithelium, ependymal, neurons, etc., accounting for about 45% of all intracranial tumors. It is generally known that glioma cells have characteristics of proliferation and migration, and finally leading to the death of patients 5-7. Nevertheless, the underlying mechanism of cell proliferation and migration in human Glioma has not been fully elucidated.

Evodiamine is one of the main effective components of Evodia rutaecarpa, which has analgesic, blood pressure lowering and tranquilizing pharmacological effects. It has been reported that Evodiamine regulates cell apoptosis, proliferation, hypertrophy and differentiation 8, 9. Evodiamine can also inhibit proliferation and promote apoptosis of tumor cells including glioma cells, indicating that Evodiamine has a certain application prospect in the clinical treatment of gliomas 10-13. In order to enhance its bioavailability and efficacy, structural optimization on Evodiamine has been carried out in our group. The derivative of Evodiamine (10-hydroxy-1-methyl-8,13b-dihydro-5H,7H-benzo[e]benzofuro[2',3':3,4]pyrido[2,1-b][1,3]oxazin-5-one) was developed by our group and named WZY-321. WZY-321 had significant cytotoxicity and could efficiently induce glioma cell apoptosis, which was superior to Evodiamine (data not shown); however, the mechanism of WZY-321-induced glioma cell apoptosis is not clear.

It is generally accepted that cell apoptosis is associated with the expression of various apoptosis-related genes such as X-linked inhibitor of apoptosis-associated factor 1 (XAF1) 14, 15. Reportedly, XAF1 functions as an antagonist of X-linked inhibitor of apoptosis (XIAP) by rescuing XIAP-suppressed caspase-3 activity, thus promotes cellular apoptosis 16-19. XAF1 expression is downregulated in a variety of tumor cells including gastric and colon cancer cell lines, and transient expression of XAF1 sensitizes tumor cells to the pro-apoptotic effects of some anti-tumor drugs 14, 17, 19-21. Reportedly, XAF1 could be epigenetically silenced in high grade gliomas 22. However, the expression of XAF1 gene in glioma cells after WZY-321 treatment and its roles in WZY-321-induced glioma cell apoptosis are unclear.

MicroRNA (miRNA) is a class of small RNA about 20-24 nucleotides. It is a single-stranded RNA precursor of about 70-90 base-sized hairpin structures processed by Dicer enzymes 23, 24. Previous studies have confirmed that miRNA can bind to the 3' UTR region mRNA of the target gene, and degrade mRNA or inhibit its protein translation. Existing studies have shown that miRNA can regulate cell apoptosis, proliferation, differentiation and other biological behavior, 24-26 and involved in the regulation of the occurrence and development of various diseases including glioma 27, 28. Notably, XAF1 might be a potential target gene miR-873 by bioinformatics analysis, suggesting that XAF1 may be negatively regulated by miR-873 at posttranscriptional level. Reportedly, miR-873 can affect cell proliferation, apoptosis and other biological processes by decreasing the expression of its target genes such as NDFIP1, Keap1 and TRIM25 29-31. So, the regulatory effect of WZY-321 on miR-873 expression and miR-873-mediated XAF1 expression in glioma cells and its mechanism need further studies.

Long non-coding RNA (lncRNA) is a non-coding RNA with a length of more than 200 nucleotides. lncRNA plays an important regulatory role in cell proliferation, apoptosis, differentiation and autophagy, and related to some types of cancers such as melanoma and breast cancer 32-35. Multiple evidences support the concept that lncRNA can interact with miRNA as a competitive endogenous RNA (ceRNA) and participate in the regulation of target gene expression 36, 37. lncRNA MTM, also known as metallothionein 1D Pseudogene (MT1DP), is located on chromosome 16 and belongs to the metallothionein family. MTM has been shown to inhibit tumor growth 38, 39; however, up to date, the expression and function of MTM in gliomas have not been reported. Our previous studies showed that MTM was significantly up-regulated in glioma cells after WZY-321 treatment, further overexpression of MTM markedly down-regulated the content of miR-873, suggesting that MTM may cause miR-873 degradation through binding to miR-873. Therefore, it is worthy to further study the roles of WZY-321 in MTM up-regulation and MTM-mediated miR-873 down-regulation in glioma cells.

## Materials and methods

### Reagents

Rabbit antibodies against XAF1 (ab17204) were purchased from Abcam (Cambridge, England). Rabbit antibodies against β-actin (4970) and HRP-conjugated anti-rabbit (7074) were purchased from Cell Signaling Technology (Danvers, USA). Enhanced chemiluminescence (ECL) HRP substrate was supplied by Thermo (Fremont, USA). The plasmid of pcDNA3.1 and Lipofectamine 2000 were purchased from Invitrogen (Carlsbad, USA). The plasmids of pGL3-promoter and pRL-SV40 luciferase reporter were purchased from Promega (Madison, WI, USA). The shRNA expression plasmids of pGPU6-GFP-Neo vector were from GenePharma (Shanghai, China). QIAprep spin miniprep kit was obtained from Biomiga (San Diego, CA, USA). An annexin V-APC/PI kit was purchased from Bender MedSystems (Vienna, Austria). Cell Counting Kit-8 (CCK-8) was form MedChemExpress (Monmouth Junction, NJ, USA).

### shRNA, miRNA and lncRNA plasmid generation

To silence human XAF1 and MTM genes, different shRNA sequences against the mRNA of XAF1 and MTM were designed. The plasmids of XAF1 shRNA (shXAF1) and MTM shRNA (shMTM) were constructed by using pGPU6-GFP-Neo, and the most effective shRNA expression plasmids were chosen for further experiments. Meanwhile, scrambled control shRNA (shCTR) expression plasmids were produced as a negative control.

All of the miR-873 mimics and a universal negative control miRNA mimic (NC mimic) were purchased from GenePharma (Shanghai, China). MTM overexpression plasmid (pcDNA3.1-MTM) was constructed.

### Cell culture and WZY-321 determination

**Figure 1:**
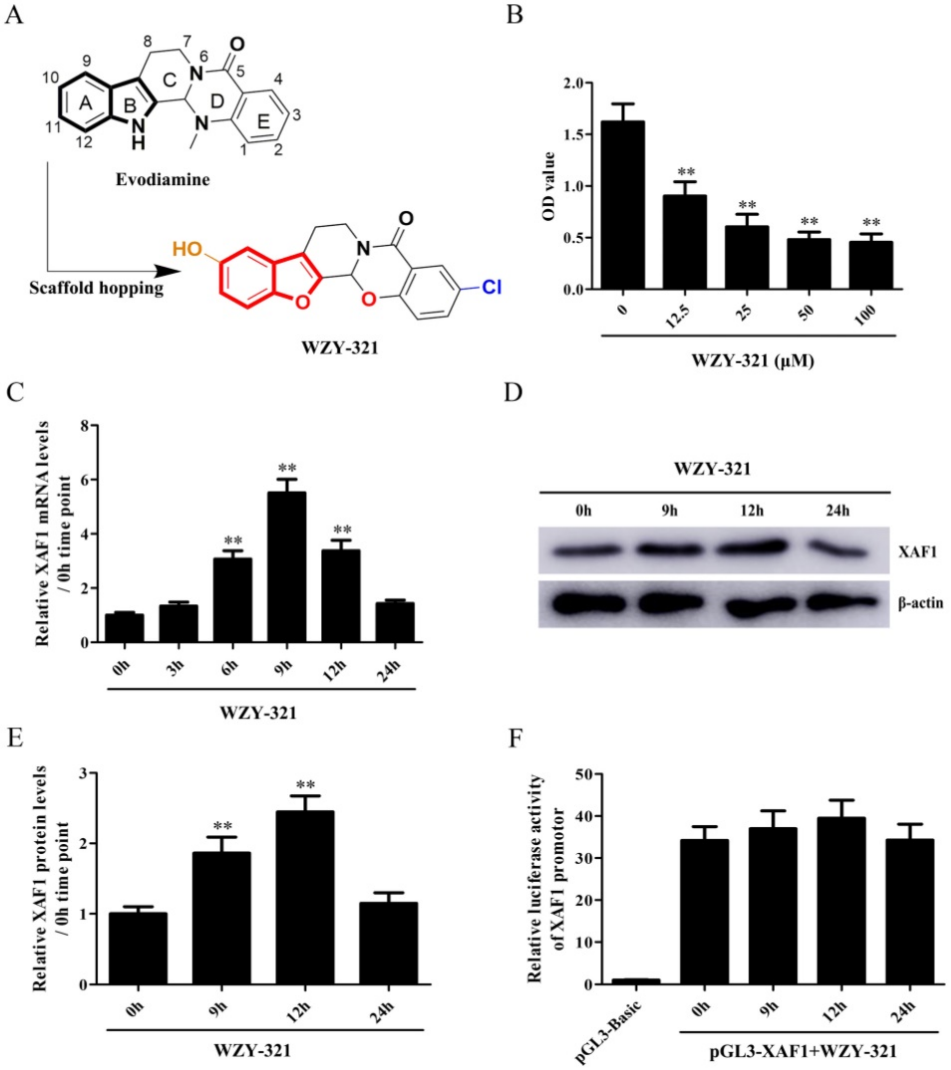
** WZY-321 increased XAF1 expression in glioma cells.** A: The molecular structure of WZY-321. B: SHG44 cells were cultured and treated with WZY-321 (0, 12.5, 25, 50, 100 µM) for 24h, and then the cytotoxicity of WZY-321 on cells was determined by CCK-8 assay. C-E: SHG44 cells were cultured and treated with WZY-321 (50 µM). The expression levels of XAF1 mRNA and protein were determined by Real-time PCR (C) and Western blotting (D and E) at different time points (0h, 3h, 6h, 9h, 12h and 24h). F: SHG44 cells were transfected with pGL3-XAF1-full-length (pGL3-XAF1-FL) or pGL3-Basic for 48h, and then treated with WZY-321 (50 µM) for another 9h, 12h or 24h. The luciferase activity was detected in different groups of cells. Results were represented as means ± SD (*n*=3 in each group). ** *P*<0.01 *versus* 0 µM or 0h group (non-treated).

The human glioma cell line of SHG-44 was obtained from Cell Bank of Chinese Academy of Sciences (Shanghai, China). Cells was cultured in DMEM supplemented with 10% (v/v) FBS and antibiotics (50 U/ml penicillin and 100 µg/ml streptomycin, Invitrogen) at 37°C in 5% CO_2_. WZY-321 was constructed by our lab and its chemical structure was shown in Fig. 1A.

### Cell transfection and identification

SHG-44 cells were transfected with shXAF1, shMTM or shCTR by using Lipofectamine 2000 according to the manufacturer's instructions.

### Real-time PCR

Total RNA was extracted from the glioma cells with TRIzol reagent. For miRNA detection, cDNA was synthesized with TaqMan microRNA reverse transcription kits. The expression level of miR-873 was assayed with TaqMan MicroRNA Assay and the TaqMan universal master mix II. The reaction program included an initial step for denaturation at 95 °C for 10 min and then 40 cycles of denaturation at 95 °C for 15 s and annealing at 60 °C for 1 min. The level of miR-873 was normalized to the level of the U6 gene.

For mRNA or lncRNA detection, cDNA was synthesized by using HisScript Ⅱ QRT SuperMix for qPCR. The expression levels of human XAF1 mRNA or MTM lncRNA were quantified by real-time PCR with AceQ qPCR SYBR Green Master Mix. Amplification of cDNA was performed on an ABI Prism 7500 (Applied Biosystems) system with a reaction program that included an initial step at 50 °C for 2 min and 95 °C for 10 min, and then 40 cycles of denaturation at 95 °C for 15 s and annealing at 60 °C for 1 min. Each sample was assayed in triplicate. Relative gene expression levels were obtained using the formula 2 ^- △△Ct^.

### Western blotting

Cells were lysed using RIPA lysis buffer. Equal quantities of protein were separated by 4-20% ExpressPlus™ PAGE Gel (Genscript, Nanjing, China) and transferred to PVDF membranes. Thereafter, PVDF membranes were incubated with primary antibodies against β-actin (1: 1000) and XAF1 (1: 1000) at 4°C overnight. After washing, PVDF membranes were incubated with HRP-anti-rabbit IgG (1: 2000) at room temperature for 40 min. Finally, the density of radiographic band onto PVDF membranes was analyzed by using the software of Quantity One (Bio-Rad, Hercules, CA, USA).

### Flow cytometry

5×10^5^ glioma cells were resuspended in binding buffer containing Annexin V-APC (1: 100) and propidium iodide (1: 100), and incubated in dark at room temperature for 15 minutes 15. The samples were analysed on a FACScan flow cytometer (BD). The percentage of apoptotic cells in a 10, 000 cell cohort was determined by flow cytometry.

### Luciferase reporter assay

The pGL3-XAF1-3'UTR was constructed by inserting the XAF1 mRNA 3'UTR into pGL3-promoter vector. For the HIF-1α 3′UTR reporter assay, SHG44 cells in each well of 24-well cell culture plates were transfected with a mixture of 0.5 μg pGL3-XAF1-3'UTR, 2.5 ng pRL-SV40 and 100 nmol miRNA mimics by using Lipofectamine 2000. Cells were lysed 48h after transfection, and then, the luciferase activity of XAF1 3′UTR reporter was measured through a dual luciferase reporter gene assay. The ratio of firefly luciferase to Renilla luciferase was calculated for each cellular sample.

### Statistical analysis

Data are presented as means ± SD. One-way ANOVA was used to determine significant differences among groups. Where significant differences were found, individual comparisons were made between groups using the t-statistic and adjusting the critical value according to the Bonferroni method. *P*<0.05 was considered significant.

## Results

### WZY-321 increases XAF1 expression in glioma cells

The cytotoxicity of WZY-321 on glioma cells was determined, and the results showed that 50 μM was the most effective dose (Fig. 1B). The levels of XAF1 expression were determined in the cultured glioma cells after WZY-321 stimulation. The time course study showed that the expression levels of XAF1 mRNA increased at 3h, peaked at 9h and then reduced at 12h (Fig. 1C), and the expression levels of XAF1 protein increased at 9h, peaked at 12h and then reduced at 24h (Fig. 1D and E). To further test whether WZY-321 can affect XAF1 gene transcription, the glioma cells were transfected with pGL3-XAF1-full-length (pGL3-XAF1-FL) for 48h, and then the glioma cells were treated with WZY-321 for another 9h, 12h or 24h. The luciferase analysis showed that WZY-321 treatment did not enhance XAF1 promotor activity in the glioma cells (Fig. 1F), suggesting that WZY-321 could increase XAF1 expression at posttranscriptional level in glioma cells.

### WZY-321 triggers glioma cell apoptosis through increasing XAF1 expression

**Figure 2:**
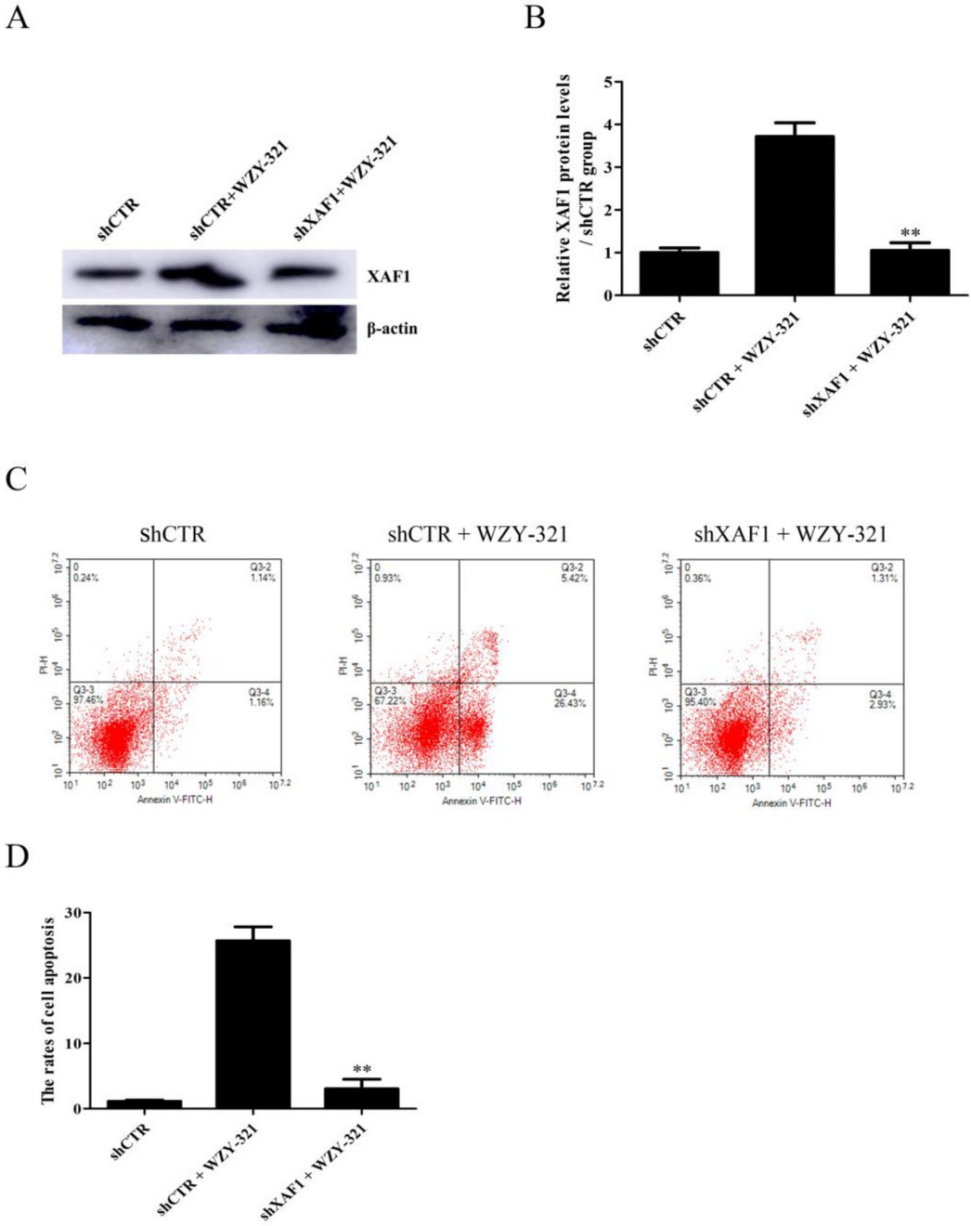
** WZY-321 induced glioma cell apoptosis through increasing XAF1 expression.** SHG44 cells were transfected with shXAF1 or shCTR for 48h, and then treated with WZY-321 (50 µM) for 12h. The XAF1 expression and cell apoptosis were examined by Western blotting (A and B) and flow cytometry (C and D). Results were represented as means ± SD (*n*=3 in each group). ** *P*<0.01 *versus* shCTR + WZY-321 group.

To explore the role of XAF1 gene in glioma cell apoptosis in response to WZY-321, the cultured glioma cells were divided into the following 3 groups, (1) shCTR, (2) shCTR + WZY-321, (3) shXAF1 + WZY-321. The studies showed that shXAF1 treatment not only reduced XAF1 expression (Fig. 2A and B) but also reduced glioma cell apoptosis (Fig. 2C and D) induced by WZY-321, indicating that WZY-321 lead to glioma cell apoptosis through up-regulating XAF1 expression.

### WZY-321 induces glioma cell apoptosis via inhibiting miR-873-induced XAF1 down-regulation

**Figure 3:**
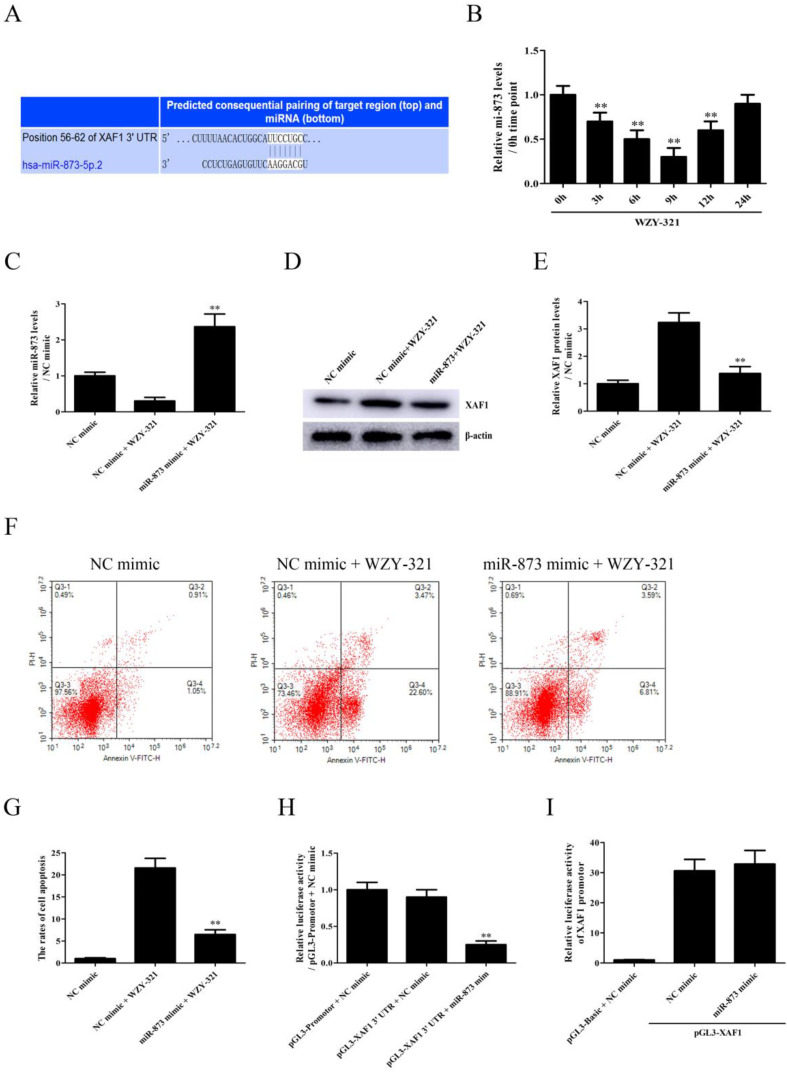
** The effects of miR-873 on XAF1 expression and glioma cell apoptosis induced by WZY-321.** A: The miR-873-binding to XAF1 3' UTR was predicted by TargetScan Release. B: The levels of miR-873 expression in SHG44 cells after WZY-321 (50 µM) treatment were determined by Real-time PCR. ** *P*<0.01 *versus* 0h group (non-treated). C-G: SHG44 cells were transfected with miR-873 mimic or NC mimic, and then incubated with WZY-321 (50 µM). The expression levels of miR-873 and XAF1 in SHG44 cells were detected by Real-time PCR (C) and Western blotting (D and E), and the apoptosis rate of SHG44 cells were detected by flow cytometry (F and G). ** *P*<0.01 *versus* NC mimic + WZY-321 group. H: SHG44 cells were transfected with different plasmids or mimic as follows: (1) pGL3-promotor + NC mimic, (2) pGL3-XAF1 3' UTR + NC mimic, (3) pGL3-XAF1 3' UTR + miR-873 mimic. Luciferase activity in different groups was determined. ** *P*<0.01 *versus* pGL3-XAF1 3' UTR + NC mimic group. I: SHG44 cells were transfected with different plasmids or mimic as follows: (1) pGL3-Basic + NC mimic, (2) pGL3-XAF1 + NC mimic, (3) pGL3-XAF1 + miR-873 mimic. The luciferase activity of pGL3-XAF1 in different groups was determined. Results were represented as means ± SD (*n*=3 in each group).

Since, it was predicted that miR-873 could bind to XAF1 3' UTR (Fig. 3A), the levels of miR-873 expression were determined in the cultured glioma cells after WZY-321 stimulation. The time course study showed that the expression of miR-873 mRNA decreased at 3h, with maximum decrease at 9h and then increased at 12h (Fig. 3B). To determine whether miR-873 down-regulation is involved in WZY-321-induced XAF1 expression in glioma cells, the cultured glioma cells were divided into 3 groups of (1) NC mimic, (2) NC mimic + WZY-321, (3) miR-873 mimic + WZY-321. As presented in Fig. 3C-G, overexpression of miR-873 by using miR-873 mimic (Fig. 3C) markedly decreased XAF1 expression (Fig. 3D and E) and glioma cells apoptosis (Fig. 3F and G) caused by WZY-321. To check whether miR-873 could bind to XAF1 3' UTR in glioma cells, the cultured glioma cells were divided into 3 groups of (1) pGL3-promotor + NC mimic, (2) pGL3-XAF1 3' UTR + NC mimic, (3) pGL3-XAF1 3' UTR + miR-873 mimic. Luciferase experiments showed that miR-873 mimic could decrease the activity of pGL3-XAF1 3' UTR (Fig. 3H). However, further experiments showed that miR-873 mimic could not affect the activity of pGL3-XAF1 (Fig. 3I). These data implicate that WZY-321 could up-regulate XAF1 gene expression and further increase glioma cells apoptosis via decreasing miR-873 expression, in which miR-873 bound to XAF1 3' UTR and decreased XAF1 mRNA levels.

### MTM-mediated miR-873 down-regulation is required for WZY-321-induced XAF1 expression and glioma cell apoptosis

**Figure 4:**
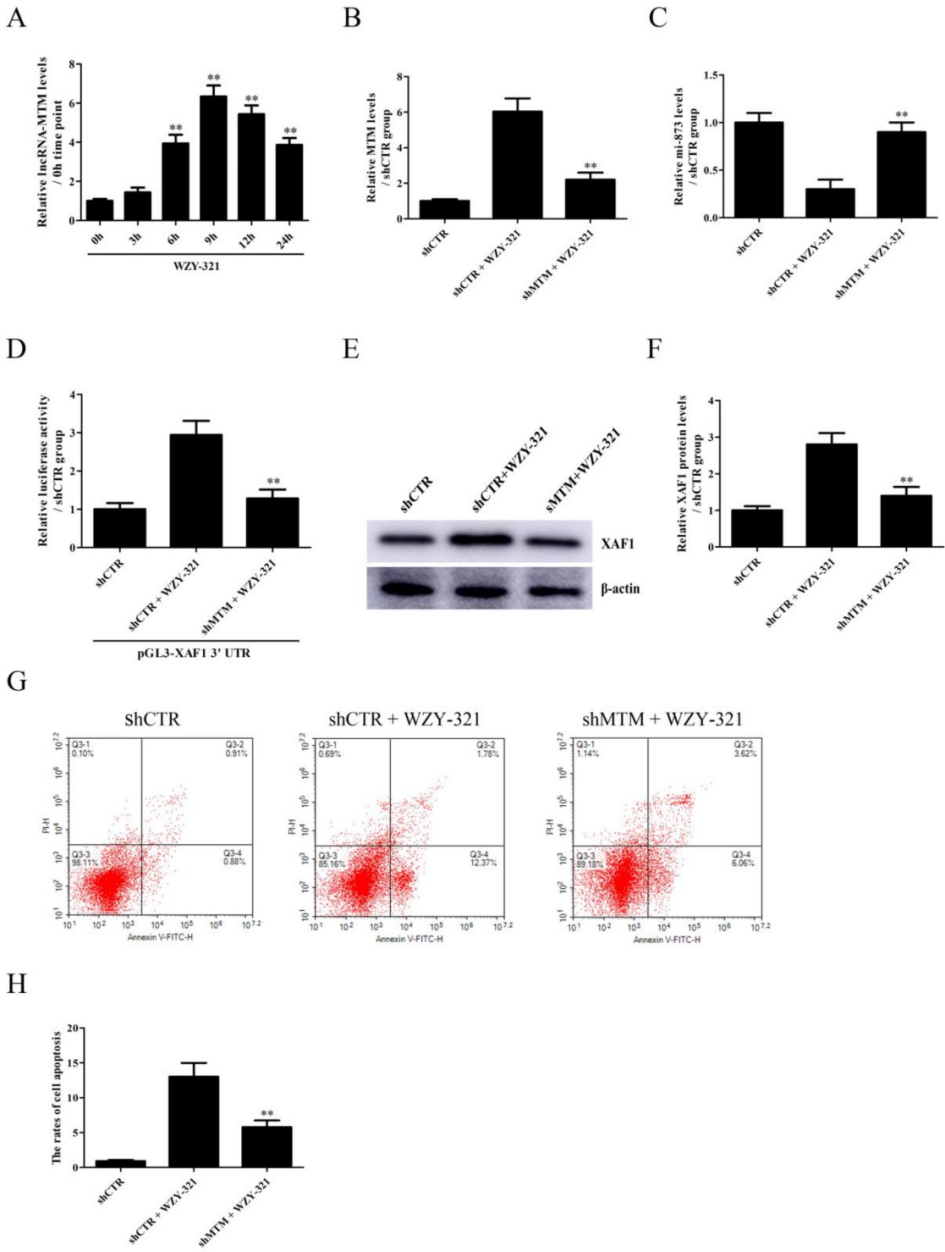
** The effects of MTM knockdown on miR-873 and XAF1 expression in glioma cells and cell apoptosis induced by WZY-321.** A: SHG44 cells were treatment with WZY-321 (50 µM) and the expression levels of MTM were determined by Real-time PCR at different time points (0h, 3h, 6h, 9h, 12h and 24h). ** *P*<0.01 *versus* 0h group (non-treated). B-G: SHG44 cells were transfected with shMTM or shCTR for 48h, and then treated with WZY-321 (50 µM) for 9h or 12h. The expression levels of MTM, miR-873 and XAF1 were examined by Real-time PCR (B and C) and Western blotting (E and F), and the apoptosis rates of SHG44 cells were detected by flow cytometry (G and H). ** *P*<0.01 *versus* shCTR + WZY-321 group. D: SHG44 cells were transfected with different plasmids as follows: (1) pGL3-XAF1 3' UTR + shCTR, (2) pGL3-XAF1 3' UTR + shCTR + WZY-321, (3) pGL3-XAF1 3' UTR + shMTM + WZY-321. Luciferase activity of pGL3-XAF1 3' UTR in different groups was determined. ** *P*<0.01 *versus* pGL3-XAF1 3' UTR + shCTR + WZY-321. Results were represented as means ± SD (*n*=3 in each group).

**Figure 5:**
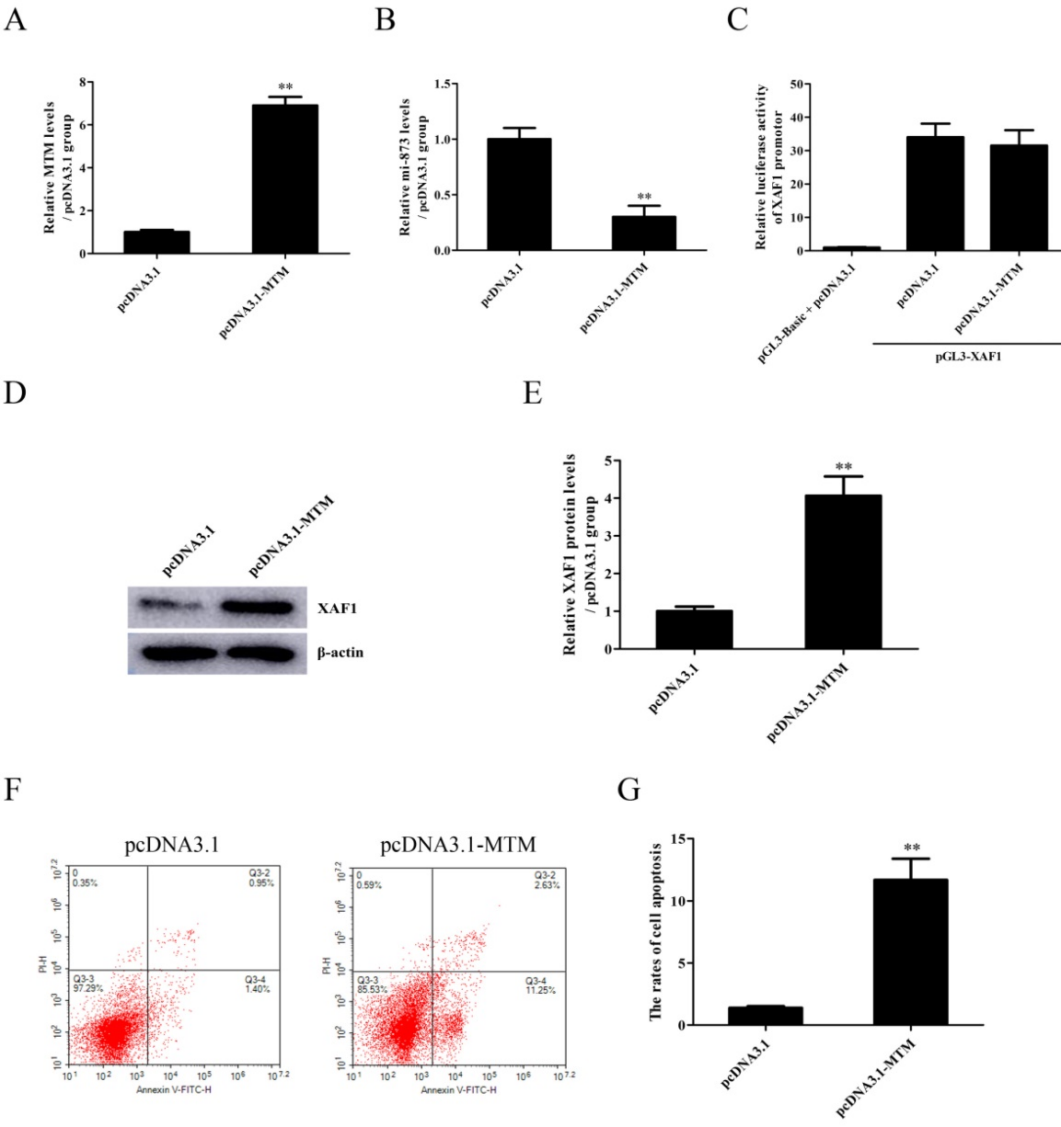
** The effects of MTM overexpression on miR-873 and XAF1 expression in glioma cells and cell apoptosis.** A-G: SHG44 cells were transfected with pcDNA3.1-MTM or pcDNA3.1 for 48h, and then treated with WZY-321 (50 µM) for 9h or 12h. The expression levels of MTM, miR-873 and XAF1 were examined by Real-time PCR (A and B) and Western blotting (D and E), and the apoptosis rates of SHG44 cells were analyzed by flow cytometry (F and G). C: SHG44 cells were transfected with different plasmids as follows: (1) pGL3-Basic + pcDNA3.1, (2) pGL3-XAF1 + pcDNA3.1, (3) pGL3-XAF1 + pcDNA3.1-MTM. Luciferase activity in different groups was determined. Results were represented as means ± SD (*n*=3 in each group). ** *P*<0.01 *versus* pcDNA3.1 group.

The expression of lncRNA MTM emerged in a time-dependent manner, with the maximum level at 9h after exposure to WZY-321 (Fig. 4A). Furthermore, MTM knockdown (Fig. 4B) could markedly increase miR-873 expression (Fig. 4C), and reduce pGL3-XAF1 3' UTR luciferase activity (Fig. 4D) and XAF1 expression (Fig. 4E and F) in glioma cells treated with WZY-321. Additionally, MTM knockdown could markedly reduce the apoptosis of glioma cells (Fig. 4G and H). In contrast, overexpression of MTM (Fig. 5A) could decrease miR-873 expression (Fig. 5B), and induce XAF1 expression (Fig. 5D and E) as well as glioma cell apoptosis (Fig. 5F and G). However, overexpression of MTM could not affect the activity of pGL3-XAF1 (Fig. 5C), suggesting that MTM might not regulate XAF1 gene transcription.

## Discussion

Evodiamine is one of the main effective components of Evodia rutaecarpa. It has been reported that Evodiamine can inhibit proliferation and promote apoptosis of tumor cells 10, 11. For example, Evodiamine promotes the apoptosis of hepatocarcinoma cells by reducing Akt phosphorylation 12. In addition, Evodiamine increases apoptosis and cell cycle arrest of glioma cells by activating JNK signaling pathway 13. In order to enhance its bioavailability and effectiveness, structural optimization of Evodiamine was carried out in our group. The derivative of Evodiamine was developed and named WZY-321. Our previous studies showed that WZY-321 had significant cytotoxicity and could efficiently induce the apoptosis of glioma cells, which was superior to Evodiamine (data not shown); however, the mechanism of WZY-321-induced glioma cell apoptosis remains unclear.

Reportedly, XAF1 functions as an antagonist of XIAP by rescuing XIAP-suppressed caspase-3 activity, leading to cellular apoptosis 16-19. XAF1 is also implicated as a tumor suppressor and is down-regulated in a variety of tumor cells including gastric and colon cancer cells, and overexpression of XAF1 sensitizes tumor cells to the pro-apoptotic effects of some anti-tumor drugs 14, 17, 19-21. It has been reported that XAF1 expression negatively correlates with the survival of patients with gliomas 40. Our current studies showed that WZY-321 increased XAF1 expression in glioma cells, and up-regulated XAF1 resulted in glioma cell apoptosis. Together, these data indicate that XAF1 up-regulation not only contributes to the glioma cell apoptosis but also enhances the sensitivity of glioma cells to anti-tumor drugs. As a result, XAF1 might be a new potential target for glioma therapy.

It is well known that miRNA can bind to the mRNA 3' UTR region of the target genes, and degrade the corresponding mRNA or inhibit its protein translation. Functionally, miRNA can regulate cell apoptosis, proliferation, differentiation and so on 23, 25, 26. Our current studies showed that miR-873 bound to XAF1 3' UTR and decreased XAF1 mRNA levels in glioma cells. WZY-321 could up-regulate XAF1 gene expression and further increase glioma cell apoptosis via decreasing miR-873 expression. Different to our finding about miR-873's function in gliomas, Wang et al reported that the overexpression of miR-873 dramatically reduced the cell proliferation, migration, and invasion of glioma cells 41. Thus, we think miR-873 expression might exhibit more complex roles in gliomas due to different cell culture conditions or different drugs.

LncRNA MTM, also known as MT1DP, is able to inhibit tumor growth 38, 39, 42. For example, MTM overexpression inhibits proliferation and induces apoptosis of hepatocarcinoma cells 38. MTM also sensitizes erastin-induced ferroptosis via regulating miR-365a-3p/NRF2 axis in non-small cell lung cancer cells 42. Overexpression of MTM in gastric cancer cells induces cellular apoptosis and inhibits cellular proliferation, migration and invasion 39. MTM could bind to microRNA and regulate cell biological behaviors. For example, MTM interacts with miR-365 and induces apoptosis of nucleus pulposus cells 43. However, up to date, the expression and function of MTM in glioma have not been reported. Our current studies showed that MTM expression was obviously up-regulated in glioma cells after WZY-321 treatment, further overexpression of MTM markedly down-regulated the expression of miR-873, suggesting that MTM may cause miR-873 degradation through binding to miR-873. These data indicate that MTM might be a new potential target for glioma therapy.
